# Crossmodal associations modulate multisensory spatial integration

**DOI:** 10.3758/s13414-020-02083-2

**Published:** 2020-07-05

**Authors:** Jonathan Tong, Lux Li, Patrick Bruns, Brigitte Röder

**Affiliations:** 1 Biological Psychology and Neuropsychology, University of Hamburg, Von-Melle-Park 11, 20146 Hamburg, Germany; 2 Centre for Vision Research, Department of Psychology, York University, Toronto, Ontario Canada

**Keywords:** Multisensory processing, Audiovisual integration, Ventriloquism effect, Causal inference, Multisensory binding, Crossmodal association, Priors, Causal prior, Coupling prior, Bayesian

## Abstract

According to the Bayesian framework of multisensory integration, audiovisual stimuli associated with a stronger prior belief that they share a common cause (i.e., causal prior) are predicted to result in a greater degree of perceptual binding and therefore greater audiovisual integration. In the present psychophysical study, we systematically manipulated the causal prior while keeping sensory evidence constant. We paired auditory and visual stimuli during an association phase to be spatiotemporally either congruent or incongruent, with the goal of driving the causal prior in opposite directions for different audiovisual pairs. Following this association phase, every pairwise combination of the auditory and visual stimuli was tested in a typical ventriloquism-effect (VE) paradigm. The size of the VE (i.e., the shift of auditory localization towards the spatially discrepant visual stimulus) indicated the degree of multisensory integration. Results showed that exposure to an audiovisual pairing as spatiotemporally congruent compared to incongruent resulted in a larger subsequent VE (Experiment 1). This effect was further confirmed in a second VE paradigm, where the congruent and the incongruent visual stimuli flanked the auditory stimulus, and a VE in the direction of the congruent visual stimulus was shown (Experiment 2). Since the unisensory reliabilities for the auditory or visual components did not change after the association phase, the observed effects are likely due to changes in multisensory binding by association learning. As suggested by Bayesian theories of multisensory processing, our findings support the existence of crossmodal causal priors that are flexibly shaped by experience in a changing world.

**Electronic supplementary material:**

The online version of this article (10.3758/s13414-020-02083-2) contains supplementary material, which is available to authorized users.

## Introduction

When talking with multiple speakers in a noisy environment, we combine the sound of a voice and the sight of moving lips to identify who is speaking, which typically increases the intelligibility of his or her words (Grant & Seitz, 2001; Schwartz, Berthommier, & Savariaux, 2004). This is an example of sensory cue integration (Trommershäuser, Kording, & Landy, 2011). A cue is a sensory signal that bears information about the state of some stimulus characteristic, such as location or identity. Integrating redundant cues regarding a common object or event has been demonstrated to reduce processing ambiguity and increase perceptual precision (Ernst & Bülthof, 2004; Rohde, van Dam, & Ernst, 2015). However, these benefits need to be weighed against the risk of integrating unrelated cues, which would in many situations lead to detrimental consequences for action. Therefore, observers need to infer the unknown causal structure that has generated the cues, thereby optimizing between seamless cue integration and appropriate segregation. This fundamental challenge is known as perceptual *causal inference* (Kayser & Shams, 2015; Shams & Beierholm, 2010). To solve this challenge, observers exploit statistical patterns across the cues and use cue correlations to infer causality (Parise, Spence, & Ernst, 2012). A statistical pattern that is particularly informative about the latent causal structure is the spatial and temporal relationships of the cues (Ernst, 2007; Parise, 2016; Spence, 2011): cues from a common origin tend to coincide or be proximate in space and time, whereas cues from different origins tend to be spatially separate and temporally uncorrelated. Therefore, it is a reasonable strategy for observers to rely on spatiotemporal patterns to determine when and to what extent different cues should be integrated. Indeed, when different cues are presented close to each other in space and time, observers are more likely to ascribe a common underlying cause and integrate the cues, while perceptual integration breaks down when the spatial or temporal discrepancies between cues exceed a certain degree (Ernst & Bülthof, 2004; Parise et al., 2012; Slutsky & Recanzone, 2001).

The perceptual endeavor of striking for a balance between cue integration and segregation has been computationally modeled with Bayesian inference, which provides a coherent framework for quantifying how an ideal observer updates his or her belief about an unknown variable in light of new observations and previous knowledge (Doya, Ishii, Pouget, & Rao, 2007). In the Bayesian framework, belief is represented by probability. Two probability components jointly determine the perceptual estimates: the likelihood function (or *likelihood* for short) and the prior probability distribution (or *prior* for short). The likelihood describes the conditional probability of observing the sensory evidence if the stimulus variable takes a certain value. For example, in a spatial localization task, a reliable stimulus would give rise to a sharp likelihood, because the sensory data it elicits would vary little from trial to trial. The prior characterizes existing experience or expectation about the sensory world prior to the new observations. Bayesian inference models of multisensory integration propose that the degree of cue integration depends primarily on two factors: One is the relative reliability of each sensory cue, which affects the relative contribution of each unisensory likelihood to the final multisensory estimate; the other is the prior belief of whether the cues are related to a common cause – known as the *causal prior* (Körding et al., 2007) – which determines the *a priori* probability for perceptually binding the cues (Odegaard, Wozny, & Shams, 2015, 2017). Here multisensory *binding* refers to the process by which information from different senses is perceived as originating from the same object or event. The Bayesian causal inference model (Körding et al., 2007) predicts that a larger causal prior (i.e., a stronger belief that two cues belong to the same event before observing these cues) would increase binding and therefore increase multisensory cue integration.

Bayesian priors in general reflect stimulus statistics in the environment (Adams, Graf, & Ernst, 2004; Angelaki, Gu, & Deangelis, 2009; Ernst, 2006; Parise, Knorre, & Ernst, 2014). Research has shown that observers can learn novel associations upon repeated exposure to statistical correlations across the cues (Ernst, 2007; Ernst & Bülthof, 2004; Flanagan, Bittner, & Johansson, 2008; Kaliuzhna, Prsa, Gale, Lee, & Olaf, 2015; Kerrigan & Adams, 2013) and use correlations to infer causal structure (Parise et al., 2012). This learning effect manifests as a modification of the subsequent tendency of spatial (Odegaard et al., 2017) or temporal (Habets, Bruns, & Röder, 2017) cue integration. To probe multisensory cue integration, the *ventriloquism effect* (VE) paradigm has often been employed as an experimental tool (Bertelson & Aschersleben, 1998). For example, in spatial ventriloquism, vision attracts sound localization, and the extent of this influence is measured as the size of the VE, which is taken as an indication of the degree of audiovisual (AV) cue integration (for reviews, see Bruns, 2019; Chen & Vroomen 2013). The VE has been found to be greater when observers report to have perceived the auditory and visual stimuli to be occurring from the same event (Wallace et al., 2004).

The goal of the present study was to identify whether and how previous experience with multiple AV stimuli specifically modulates their subsequent spatial integration. Unlike previous studies of the binding tendency, which tested only one pair of AV stimuli (e.g., Odegaard et al., 2017), we aimed to explore whether it was possible to simultaneously drive the crossmodal causal priors (hereafter referred to as “priors”), and therefore the degree of multisensory integration, in opposite directions for different pairs of AV stimuli. We applied a spatial VE paradigm as an indicator of the degree of AV integration following an association-learning phase, during which participants were repeatedly exposed to spatiotemporally congruent and incongruent AV pairings. The congruent AV pair was always presented at the same time and location, whereas the incongruent AV pair was presented with large spatiotemporal discrepancies. We predicted that the congruent versus incongruent pairings would drive the prior to be different for the congruent and incongruent AV pairs, resulting in different degrees of AV integration and therefore different sizes of VE. Two experiments were conducted. Experiment 1 tested whether spatiotemporally congruent versus incongruent pairings have differential effects on the sizes of the subsequent VE without changing the unisensory reliabilities of the auditory or visual components. Such a result would provide experimental evidence for the role of adaptive priors in multisensory integration. Experiment 2 tested the specificity of such newly acquired priors by flanking the auditory stimulus with two competing visual stimuli and measuring the resulting net VE. Together, the two experiments examined the flexibility and selectivity of the use of prior knowledge about the spatiotemporal relation of auditory and visual stimuli for multisensory integration.

## Experiment 1

We employed simple auditory and visual stimuli – sine tones of different frequencies and brief flashes of different colors, respectively – to avoid any semantic associations between the stimuli (Thelen & Murray, 2013). During the association phase, participants were repeatedly exposed to pairs of a tone and a flash that were either consistently aligned in both space and time (“congruent pairs”) or spatially separated and temporarily uncorrelated (“incongruent pairs”). Importantly, the specific tone and flash used for congruent versus incongruent pairs were counterbalanced across participants. Following the association phase, we measured the sizes of VE for the congruent and incongruent pairs, as well as for new pairings of the same AV components (“recombined pairs”). We predicted that greater AV integration, indicated by larger VE, would be observed for congruent pairs compared to incongruent pairs. Moreover, in order to rule out the possibility that differences in the degree of AV integration were due to changes in unisensory (auditory or visual) reliabilities or spatial biases, we compared unisensory localization precision before and after the association phase.

### Methods

#### Participants

A total of 16 healthy adults recruited from the University of Hamburg participated in the first experiment. A previous study conducted in our laboratory using a simultaneity judgment task had demonstrated that AV association learning enhanced subsequent AV temporal binding; this study had 14 participants and found moderate-to-large effect sizes (Cohen’s d ≥ 0.71) (Habets et al., 2017). *A priori* power analysis using d = 0.71 indicated that with a paired-samples design, a sample size of 16 participants would be sufficient to detect a moderate-to-large effect of AV association on the size of VE, with a power of 0.8 and an alpha of 0.05 (G*Power 3.1; Faul, Erdfelder, Lang, & Buchner, 2007). One participant was later disqualified due to poor performance on the Association blocks (see Association blocks section); data reported were based on the remaining 15 participants (age: 19–69 years, median age = 25 years, interquartile range = 21.75–29.25 years, only one individual was over 45 years old; all were right-handed, seven female). All participants were free of neurological disorders and auditory or visual deficits (corrected-to-normal vision was permitted) as determined by self-report in a pre-test questionnaire. Handedness was determined by a questionnaire based on the Edinburgh Handedness Inventory (Oldfield, 1971). Participants were reimbursed with course credits or 7€ per hour for their participation in the study. Written informed consent was obtained from each participant prior to the experiments. The experimental procedure was approved by the ethics commission of the Faculty for Psychology and Human Movement Science at the University of Hamburg, and the study was carried out in accordance with the 2013 Declaration of Helsinki.

#### Apparatus and stimuli

Participants were seated comfortably in the center of a semicircular array of six loudspeakers (ConceptC Satellit, Teufel GmbH, Berlin, Germany) located at ± 4.5°, ± 13.5°, and ± 22.5° relative to the center (negative values: left to the center), at a height roughly equal to eye level and a distance of 90 cm from the participant’s head. A chin rest held the participant’s head in a fixed position, directly facing forward, towards the speakers. An acoustically transparent black curtain, extending the full 180° of the semicircle, occluded the speakers. The auditory stimulus was a sine tone of either 750 Hz or 3,000 Hz with a duration of 200 ms (including 5 ms linear rise/fall envelops), presented at 65 dB(A) as measured at the participant’s head position. The visual stimulus was either a red or a blue laser flash projected onto the black curtain at the level of the speakers for a duration of 200 ms. The laser pointers were attached to step motors that allowed projection at different azimuthal locations. The luminance of the laser flashes was low enough so that the participants could only see the flash but not the details of the experimental setup. During test trials, the participant used a pointing stick, mounted on a potentiometer below the chinrest, to indicate the perceived location of the stimulus, pushing a button attached to the stick to register each response. Participants were instructed to use both hands to reposition the stick.

#### Testing procedure

**Fig. 1:**
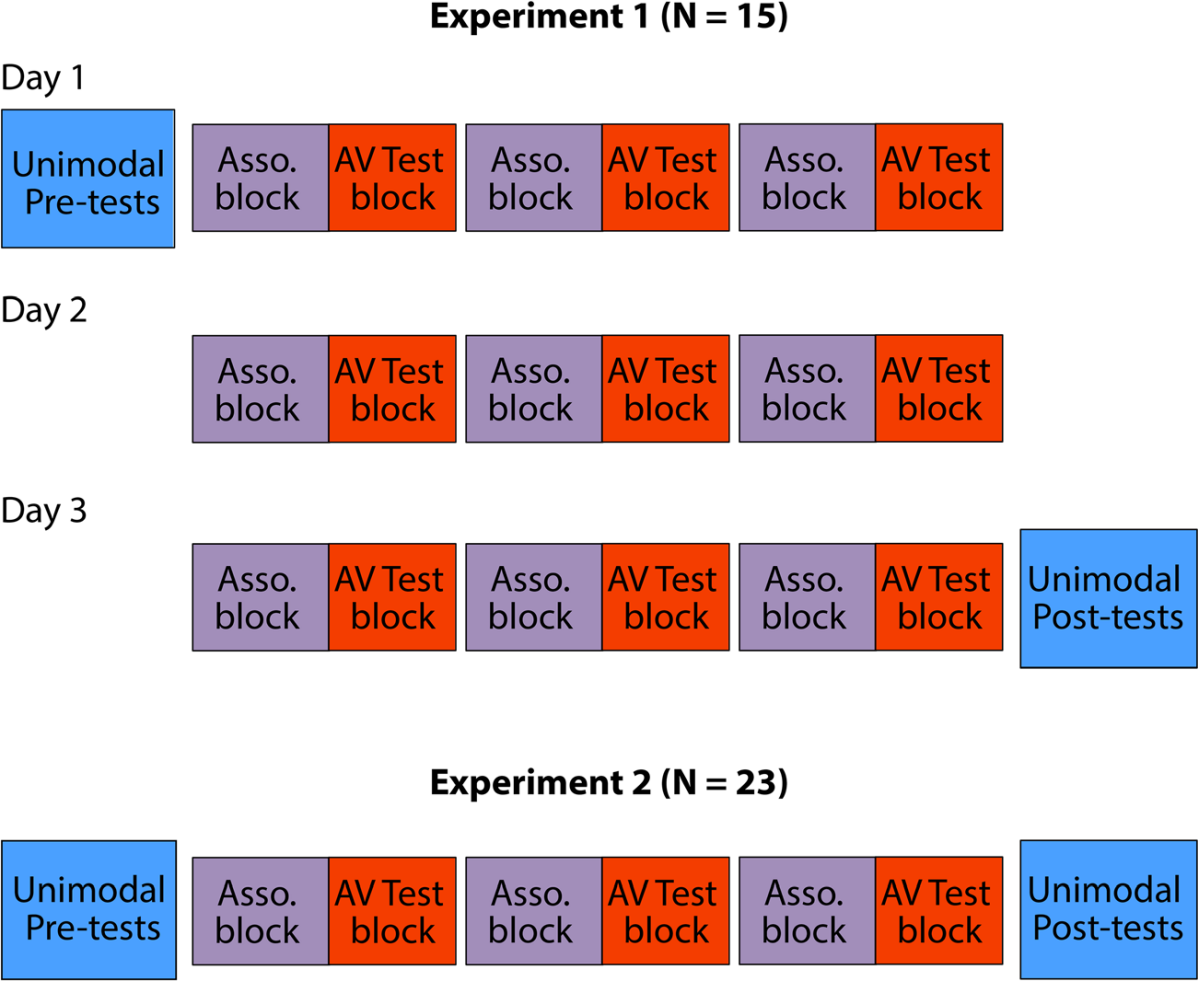
Overall testing procedures for Experiments 1 and 2. **Top:** Experiment 1. Three testing days, each consisting of three alternating Association blocks and audiovisual (AV) Test blocks. The first and the last testing days had unimodal auditory (A) and visual (V) localization blocks at the beginning (Pre-tests) and the end (Post-tests), respectively, to track any possible changes in unimodal reliability or spatial biases over the course of the entire experiment. **Bottom**: Experiment 2. The same testing order as Experiment 1, but only for 1 day

**Fig. 2.**
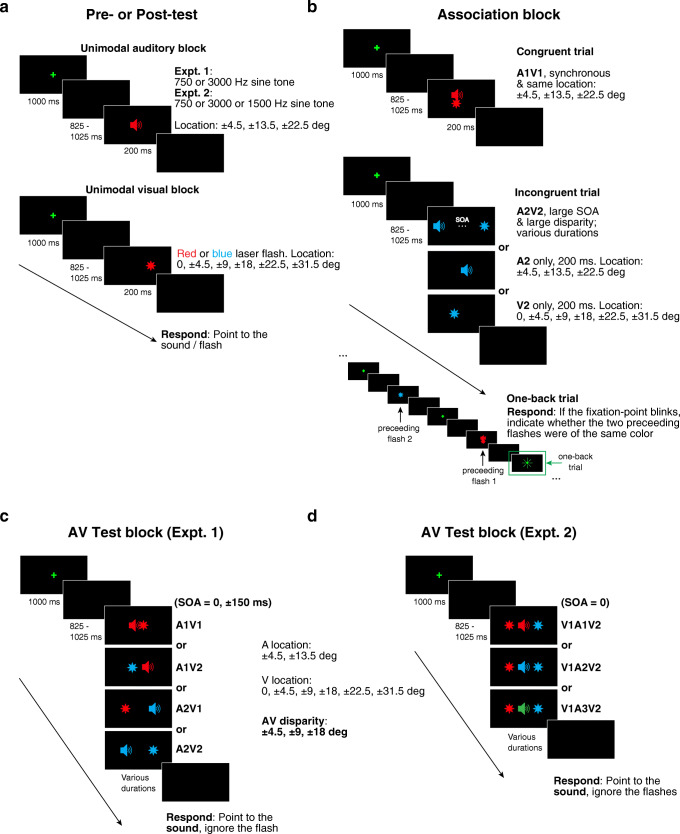
Block designs. (**a**) Unimodal Pre- or Post-test (Experiments 1 and 2). Auditory (A) and visual (V) localization was tested in separate blocks, with counterbalanced order. (**b**) Association block (Experiments 1 and 2). A mixture of congruent, incongruent, and one-back trials. Participants only responded to the intermittent and rare one-back trials, which were included to ensure that participants were paying attention to the stimuli during the Association phase. The congruent pair A1V1 were presented at the same location in the experiments; the small vertical offset in the figure is only for display clarity purpose. Three types of stimuli were applied for incongruent trials: A2V2 (bimodal), A2 only, or V2 only. A2V2 was temporally separated by a large stimulus-onset asynchrony (SOA) randomly chosen from 750 to 1,500 ms, and spatially separated at a large disparity of ± 13.5°, ± 22.5°, ± 31.5°, or ± 40.5°. (**c**) Audiovisual Test block (Experiment 1). (**d**) Audiovisual Test block (Experiment 2). V1 and V2 flanked the auditory stimulus with equal discrepancies (4.5°, 9°, or 18°), and the sides that V1 and V2 appeared were counterbalanced. The A location, V location, and AV disparity values between (**c**) and (**d**) are for both figures (the figures only illustrate some of these values). For illustration purpose, in (**b**), (**c**), and (**d**) red is used to represent the congruent stimuli (“red” sound: A1; “red” flash: V1) and blue to represent the incongruent stimuli (“blue” sound: A2; “blue” flash: V2). In the experiments, the tone frequencies and flash colors used for the congruent pair (A1V1) versus the incongruent pair (A2V2) were counterbalanced across participants and consistent for an individual participant. “Green” sound in (**d**): a novel tone A3 (1,500 Hz sine tone), never presented during Association blocks. Positive location: stimulus on the right of the screen center. Negative location: stimulus on the left of the screen center. Positive AV disparity: V on the right of A. Negative AV disparity: V on the left of A. Positive SOA: V-leading. Negative SOA: A-leading

Each participant completed three experimental sessions (Fig. 1 – Experiment 1), each session on a separate day (with the exception of one participant who completed two sessions on the same day). The number of days between two testing sessions ranged from 0 to 22, with a mean of 5.5 days. Each experimental session lasted about 2.5–3 h, including short breaks (2–3 min) roughly every 30 min. On Day 1, participants first completed two unimodal Pre-tests in which they localized either auditory (A) or visual (V) stimuli alone in separate blocks. This was followed by an Association block, in which spatiotemporally congruent and incongruent A and V stimulus pairs were presented (see Association blocks for detail) with the intention of differentiating associations between the stimuli. The Association block was followed by a Test block in which congruent, incongruent, and new pairings of the A and V components were presented (see Audiovisual (AV) Test blocks (Fig. 2c) for detail) to assess the level of multisensory integration by measuring the size of the VE. The Association and Testing blocks were run in alternating order until a total of three blocks each were completed. On Day 2, participants again completed three sets of alternating Association and Testing blocks but without doing any unimodal test blocks. On Day 3, participants completed the three sets of alternating Association and Testing blocks, followed by two unimodal (A, V) Post-tests that were identical to the unimodal Pre-tests.

The start of each trial was indicated by a green laser fixation point for 1,000 ms at the center of the black curtain at the speaker height, followed by a randomly varying delay between 825 and 1,025 ms, and then the presentation of a stimulus or stimuli (A alone, V alone, or a AV pair) (Fig. 2). The type of response depended on the types of block and trials (see corresponding block sessions below).

##### Unimodal Pre- and Post-tests (Fig. 2a)

Unimodal Pre- and Post-tests were carried out to assess the baseline unimodal localization performance of each participant. Auditory and visual localizations were tested in separate blocks, and the order of modality tested was counterbalanced across participants. The A stimulus (a 750- or 3,000-Hz sine tone) was presented pseudo-randomly at one of the six speakers (± 4.5°, ± 13.5°, and ± 22.5° relative to the center) for a total of 20 trials at each speaker location for each tone. The V stimulus (a red or blue laser flash) was presented pseudo-randomly at one of 11 locations (0°, ± 4.5°, ± 9°, ± 18°, ± 22.5°, and ± 31.5° relative to the center), also for a total of 20 trials at each location for each color. These 11 locations covered all the V stimulus locations used in the following AV Test blocks (described below in Audiovisual Test blocks), so that we were able to measure the visual reliability across the field and keep track of any reliability changes due to the association learning procedure. Immediately after the stimulus presentation, the participant pointed to the estimated stimulus location by using the pointing stick and button press.

##### Association blocks (Fig. 2b)

Each Association block consisted of randomly intermixed 384 association trials and 48 one-back trials (see One-back trials below), and lasted approximately 25 min.

i) Association trials

The association trials exposed participants to AV stimulus pairs with congruent and incongruent spatiotemporal patterns, with the goal of implicitly forming causal relationships (associations) between specific AV stimulus pairs. Each Association block consisted of 192 trials for the congruent pair and 192 trials for the incongruent pair. The congruent pair consisted of a tone and a flash that were presented synchronously at the same location, which was randomly chosen from the six speaker locations (± 4.5°, ± 13.5°, and ± 22.5° relative to the center) until 32 trials were reached for each location. The incongruent pair consisted of a tone and/or a flash that were spatiotemporally misaligned. In half of the incongruent trials (96 trials), the tone and the flash were separated by large spatial and temporal discrepancies (“bimodal incongruent trials”). The stimulus-onset asynchrony (SOA) was randomly chosen from 750 ms to 1,500 ms. Half of these bimodal incongruent trials were auditory-leading (denoted by negative SOA values), and the other half were visual-leading (denoted by positive SOA values). In the other half of the incongruent trials (96 trials), the tone or the flash was presented alone (“unimodal incongruent trials,” including 48 A-only trials and 48 V-only trials).

For all the incongruent trials, the A was presented at any of the six speaker locations: ± 4.5°, ± 13.5°, and ± 22.5° relative to the center. For the unimodal incongruent V-only trials, the V was presented at any of eight locations: ± 9°, ± 18°, ± 27°, and ± 36° relative to the center. For the bimodal incongruent trials, the V was presented at one of eight disparities relative to the A: ± 13.5°, ± 22.5°, ± 31.5°, or ± 40.5° (negative disparities mean that V was to the left of A). Here and in the rest of this paper, the term “location” refers to the *absolute* A or V location, relative to the center point of the visual field; the term “disparity” refers to the angular separation between the AV pair, i.e., the V location *relative* to the A location. The stimulus locations and AV disparities were predetermined and presented on an equal number of trials in random order. The use of large AV spatial disparities in the bimodal incongruent trials was to emphasize the signaling of spatial incongruence. The use of a wide variety of V locations was to avoid confounding systematic biases, because if participants noticed that the flash always occurred at a fixed location, they might just point to that location as their answer.

To concisely refer to the different stimulus components and pairs, the following naming system is used: A1 and V1 refer to the stimuli used in the congruent pair, and A2 and V2 refer to the stimuli used in the incongruent pair, regardless of which specific tone or flash they are. For example, for a quarter of participants, the congruent pair consisted of a 750-Hz tone (A1) and a red flash (V1), and the incongruent pair consisted of a 3,000-Hz tone (A2) and a blue flash (V2); for a different quarter of participants, the congruent pair consisted of a 750-Hz tone (A1) and a blue flash (V1), and the incongruent pair consisted of a 3,000-Hz tone (A2) and a red flash (V2). A1, A2, V1, and V2 were consistent for each individual participant and counterbalanced across participants.

ii) One-back trials

Participants performed a one-back task during the Association blocks in order to ensure that they paid attention to the stimuli and were able to discriminate the laser colors. A one-back trial was indicated by a blinking instead of a static fixation point. Participants were instructed to respond to such trials by pushing one of two buttons on a button box to indicate whether the visual stimuli in the two previous trials were of the same or of different colors. Because the two preceding visual stimuli could have occurred at a variety of locations across the experimental visual field at a variety of intervals, performance on the one-back trials required attention to space and time as well as color. Forty-eight one-back trials were randomly placed throughout the Association block with equal trials of A1V1 and A2V2. To ensure that the participants were able to pick up on these cues for forming AV associations, a > 60% accuracy on the one-back trials was required to qualify for the following tests. One of the 16 participants failed to meet this criterion, and their data were excluded from further analysis.

##### Audiovisual (AV) Test blocks (Fig. 2c)

Following each Association block, an audiovisual Test block was carried out to assess the level of AV integration. Using a typical VE paradigm, auditory localization was tested in the presence of a disparate visual stimulus. Participants were explicitly instructed to pay attention only to the auditory stimulus while maintaining fixation and to respond by localizing only the auditory stimulus. Following the offset of the fixation laser, after a short interval randomly selected from 825 to 1,025 ms, one of four possible AV pairs was presented as the test stimulus: the congruent (A1, V1) and the incongruent (A2, V2) pairs from the Association block, as well as two new combinations (A1, V2) and (A2, V1).

The A stimulus was presented at one of four possible speaker locations: ± 4.5° and ± 13.5° relative to the center. We tested four instead of six locations (i.e., excluding ± 22.5°) to ensure large numbers of trials for each location without lengthening the already long experimental duration (2.5–3 h). The V stimulus was presented at 11 possible locations: 0°, ± 9°, ± 18°, ± 22.5°, and ± 31.5° relative to the center. These locations yielded six AV spatial disparities: ± 4.5°, ± 9°, and ± 18° (negative disparity: V on the left of A; positive disparity: V on the right of A). We used a wide variety of AV spatial disparities to prevent adaptation to a specific disparity which could have led to systematic biases. Moreover, to examine whether the association effects (if observed for synchronous AV pairs) would generalize to asynchronous AV pairs, we tested three different SOA conditions: (1) simultaneous presentation of A and V (SOA = 0 ms), (2) V leading by 150 ms (SOA = 150 ms), and (3) A leading by 150 ms (SOA = −150 ms). Together, there were a total of 288 unique combinations of AV pairing, AV disparity, and SOA. Over the course of Experiment 1, each of these combinations was tested 12 times for a total of 3,456 test trials, which were evenly divided into nine Test blocks over three experimental days. In addition to these test trials, each Test block included 29 deviant trials to ensure that participants kept their eyes open during the test. The deviant trials began with a blinking fixation-point at the center, and the participants were to respond by pressing either of two buttons (the same buttons used in Association blocks). These deviant trials were later excluded from data analysis.

#### Data analysis

All statistical analyses were conducted in R version 3.5.1 with an alpha level of .05. Repeated-measures ANOVAs were performed using the R-package ‘ez’ with type III sum of squares. For effect sizes, the generalized Eta-squared (η_G_^2^) values (Bakeman, 2005) are reported. In case of violation of sphericity, Greenhouse-Geisser-corrected p-values and epsilon (ε) values are reported. For post hoc multiple comparisons (one-sample t-tests), Bonferroni-Holm correction was used to control for the familywise error rate, and the adjusted *p*-values are reported.

##### Unimodal Pre- and Post-tests

In the Pre- and Post-tests, we calculated the Constant Error and the Variable Error for unimodal auditory and visual localization for each participant. The Constant Error was a measure of localization accuracy; it was calculated as the difference between the perceived auditory stimulus location and the veridical location (perceived – veridical). The Variable Error was a measure of localization reliability; it was calculated as the standard deviation of the localization responses. The Constant Error and the Variable Error were first calculated for each stimulus location separately, and then averaged across stimulus locations, for each stimulus type (A1, A2, V1, V2), test (Pre, Post), and participant.

In order to rule out the possibility that congruent or incongruent pairing (A1V1 or A2V2) could change the reliability of the unimodal (auditory or visual) stimulus, we carried out separate 2 × 2 ANOVAs for each of the tested modalities, with measuring time (Pre-test, Post-test) and stimulus type (A1 and A2 for auditory, V1 and V2 for visual) as the two factors, and the mean Constant Error or mean Variable Error as the dependent variable. A significant interaction between the Pre-/Post-tests and the stimulus type would indicate that congruent or incongruent pairing during the Association blocks might have differentially modulated the reliabilities of the unimodal component stimuli (A1, V1, A2, and V2).

##### Association blocks

We calculated the percentage of correct responses across Association blocks for each participant’s performance on one-back trials. The data from any participant who performed below 60% were excluded from analysis, since it is important in our design that participants were paying attention to the stimuli during Association blocks and that participants were able to discriminate the different stimulus types.

##### Audiovisual Test blocks

The overall extent of the VE was derived as a ventriloquism effect index (*VE index*): For each participant and each combination of veridical A location, AV spatial disparity, stimulus pair, and SOA conditions, the mean perceived auditory location was calculated; then, for each absolute value of AV disparity (4.5°, 9°, or 18°), the mean perceived auditory location for the negative-disparity trials (i.e., V on the left, A on the right) was subtracted from the mean perceived auditory location for the positive-disparity trials (i.e., V on the right, A on the left). For example, the mean perceived auditory location for the −9° disparity trials (in which V was 9° to the left of A) was subtracted from that for the +9° disparity trials (in which V was 9° to the right of A). This difference between the corresponding means of the negative- and positive-disparity trials was taken as the ventriloquism effect index (*VE index*). If AV integration was absent, then whether V was on the left or right of A should have no impact on the perceived auditory location, which would result in a VE index value of zero; therefore, the VE index reflects the overall extent of AV integration. The VE index values were then averaged over the veridical auditory locations to provide a summary of the strength of the VE for each combination of absolute disparity (4.5°, 9°, and 18°), stimulus pair, and SOA, for each participant. The mean VE index values were analyzed with a 3 × 2 × 2 × 3 ANOVA, with Disparity (4.5°, 9°, and 18°), Auditory stimulus (A1, A2), Visual Stimulus (V1, V2), and SOA (−150, 0, and 150 ms) as factors.

### Results

#### Unimodal Pre-and Post-tests

We calculated Variable Error and Constant Error in unisensory localization performance in the Pre- and Post-tests (see Fig. S1, Supplemental Online Material). Variable Error and Constant Error are a measure for reliability and accuracy, respectively, with smaller values indicating better reliability or accuracy. Repeated-measures ANOVAs showed that measuring time (Pre-test, Post-test) or stimulus type (A1 and A2 for auditory, V1 and V2 for visual) did not have significant main effects or interactions on either Variable Error or Constant Error (all p-values ≥ .077; see Table S1, Supplemental Online Material, for details). This indicates that the association pairing (congruent vs. incongruent) manipulations did not significantly alter the reliabilities or localization accuracies of the unimodal component stimuli (A1, A2, V1, and V2). Therefore, any differential effects of association pairing on subsequent multisensory perception were unlikely to be due to changes in unisensory processing of the component stimuli.

#### Association blocks

A total of 432 one-back trials were randomly displayed throughout the Association blocks to ensure that participants were paying attention to the stimuli as well as to assess their ability to discriminate the different flash colors. All 16 participants were able to perform the one-back trials above chance levels (>50%; minimum: 54%, maximum: 89%, median: 85%), but one of them had an accuracy of 54%, which was below our criterion of 60%, and all their data were therefore excluded from further analysis (see section Participants in Methods).

#### Audiovisual Test blocks

**Fig. 3:**
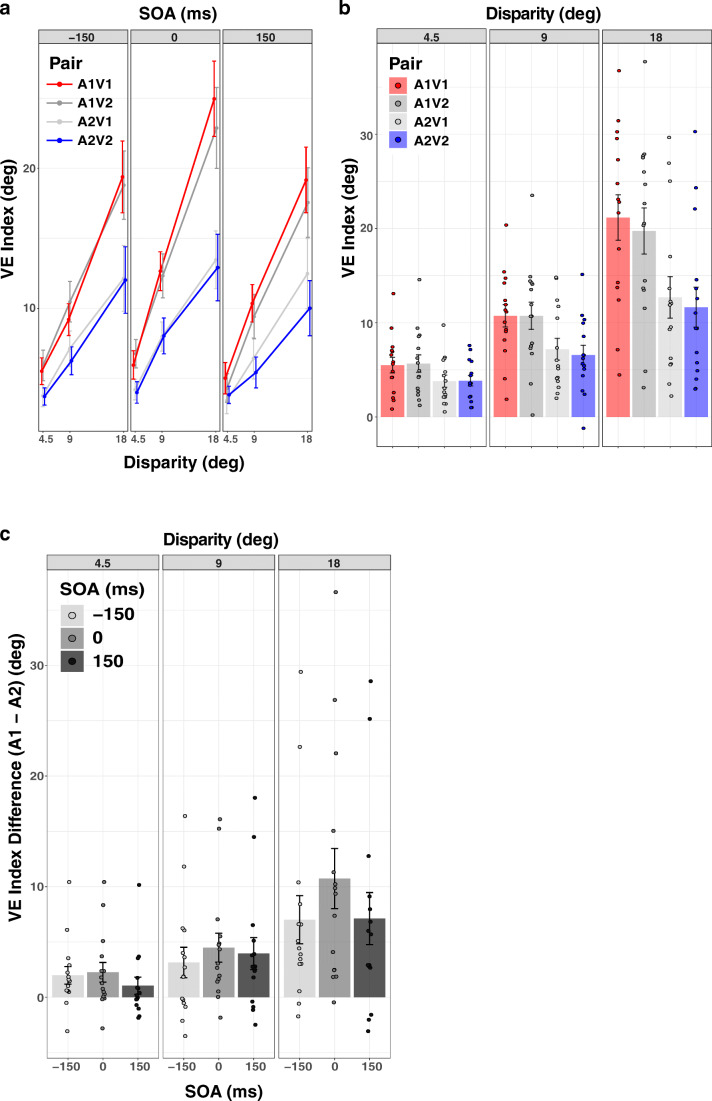
Ventriloquism effect (VE) data (Experiment 1). (**a**) VE index plotted as a function of audiovisual disparity for different stimulus-onset asynchronies (SOAs). (**b**) VE index averaged across SOAs, plotted for different audiovisual pairs. (**c**) VE index differences between A1 and A2 (VE_A1_-VE_A2_) averaged across V1 and V2, plotted for different SOAs. Dots: individual participants. Bars: condition means. Error bars: ± 1 SEM. Negative SOA: auditory first. Positive SOA: visual first. For illustration, lines and dots are slightly dodged horizontally to reduce overlap. Colors in (**a**) and (**b**) represent different AV pairs. Red: A1V1 (congruent). Dark gray: A1V2 (recombined). Light gray: A2V1 (recombined). Blue: A2V2 (incongruent)

Repeated-measures ANOVA on the VE index showed significant main effects of Auditory stimulus (F(1,14) = 10.631; p = .006, η_G_^2^ = .128), Disparity (F(2,28)= 60.151; p < .001, ε = .551, η_G_^2^ = .369), and SOA (F(2,28) = 9.634; p < .001, η_G_^2^ = .026), as well as a significant Auditory stimulus by Disparity interaction (F(2,28) = 13.142, p = .002, ε = .552, η_G_^2^ = .045), Auditory stimulus by SOA interaction (F(2,28) = 6.231, p= .006, η_G_^2^ = .004), SOA by Disparity interaction (F(4,56)=3.780, p = .027, ε = .586, η_G_^2^ = .006), and Auditory stimulus by SOA by Disparity three-way interaction (F(4,56) =3.663, p = .010, η_G_^2^ = .003). No significant main effect (F(1,14) = 2.463, p = .139, η_G_^2^ = .001) or any interaction involving the factor Visual stimulus was observed (Visual × Disparity: F(2,28) = 2.563, p = .095, η_G_^2^ = .002; Visual × SOA: F(2,28)= 1.012, p = .376, η_G_^2^ = .001; Visual × Auditory × Disparity: F(2,28)= .635, p = .538, η_G_^2^ < .001; Visual x Auditory x SOA: F(2,28) = .592, p = .489, ε = .630, η_G_^2^ < .001; Visual × Disparity × SOA: F(4,56) = .345, p = .846, η_G_^2^ < .001; Visual × Auditory × Disparity × SOA: F(4,56) = 1.354, p = .261, η_G_^2^ = .001). These results indicate that regardless of the visual stimulus, overall the perceived location for the congruent auditory stimulus A1 was more susceptible to visual influence compared to the incongruent stimulus A2 (Fig. 3a; for the mean data plotted for each condition and speaker location, see Fig. S2, Supplemental Online Material). Moreover, this auditory-based differential effect generalized to different visual stimuli (V1 and V2): the congruent pair A1V1 and the recombined pair A1V2 (i.e., the pairs including A1) led to comparable VEs, which were both greater than the VEs elicited by both the incongruent pair A2V2 and the recombined pair A2V1 (i.e., the pairs including A2). Here, it is important to note that this auditory-based differential effect could not be due to the particular tone used as A1 or as A2, because the tones employed as A1 and as A2 were counterbalanced across participants.

Next, we averaged the VE index values across SOAs for each stimulus pair at each AV spatial disparity (Fig. 3b). At larger disparities, differences in VE index between the stimulus pairs became more apparent, and the perceived auditory location shifted more towards the visual stimulus. This is consistent with findings in the literature, which have shown that the size of the spatial VE increases with AV spatial disparity up to 20–25° of separation (Bertelson & Radeau, 1981; Hairston et al., 2003; Wallace et al., 2004)

Given that we observed no significant effects of Visual stimulus but a significant main effect of Auditory stimulus and a significant Auditory-stimulus-by-Disparity interaction, we next averaged the VE index values across different visual stimuli to further explore this interaction (Fig. 3c). We conducted a 3 (SOA levels) × 3 (Disparity) repeated-measures ANOVA on the mean VE index differences between A1 and A2 (i.e., VE_A1_-VE_A2_). Results showed main effects of SOA (F(2,28) = 6.231; p = .006, η_G_^2^ = .018) and Disparity (F(2,28) = 13.142; p = .002, ε = .552, η_G_^2^ = .157), as well as a significant SOA by Disparity interaction (F(4,56)= 3.663, p = .010, η_G_^2^ = .012). The VE differences between A1 and A2 were most prominent when the auditory and visual test stimuli were presented simultaneously (i.e., when SOA = 0) at a larger spatial disparity. In addition, we conducted one-tailed t-tests comparing (VE_A1_–VE_A2_) with zero. Results showed that VE_A1_ was significantly greater than VE_A2_ at every spatial disparity (4.5, 9, 18°) and SOA levels (−150, 0, 150 ms) except at 4.5° disparity when the visual stimulus occurred 150 ms before the auditory stimulus (4.5°, −150 ms: t(14) = 2.500, p = .016, Cohen’s d = .645; 4.5°, 0 ms: t(14) = 2.532, p = .016, d = .654; 4.5°, 150 ms: t(14) = 1.347, p = .100, d = .348; 9°, −150 ms: t(14) = 2.285, p = .022, d = .590; 9°, 0 ms: t(14) = 3.406, p = .010, d = .879; 9°, 150 ms: t(14) = 2.733, p = .015, d = .706; 18°, −150 ms: t(14) = 3.210, p = .010, d = .829; 18°, 0 ms: t(14) = 3.941, p =.007, d = 1.018; 18°, 150 ms: t(14) = 3.021, p = .010, d = .780).

An explorative analysis showed that the testing day (“Day”) did not have a significant effect on the VE (F(2, 28) = .484, p = .621, η_G_^2^ = .005). In addition, Day did not significantly interact with any other independent variables (Day × A × V × SOA: F(4, 56) = 2.491, p = .053, η_G_^2^ = .001; all other Fs ≤ 1.978, all other ps ≥ .110).

### Discussion

Experiment 1 investigated whether and how a newly acquired audiovisual association or dissociation modulates the degree of audiovisual integration. We measured the spatial VE – visual influence of the perceived sound location – to probe the degree of AV spatial integration following an association-pairing phase. Results suggested that exposure to pairings of AV stimuli as either spatiotemporally congruent or incongruent had differential impacts on the subsequent VE. Specifically, exposure to a sound that was spatiotemporally misaligned with a visual stimulus resulted in a significantly smaller subsequent VE for that sound, compared to a sound that was consistently aligned in space and time with a visual stimulus. This effect generalized at least partially to AV stimuli that were never paired during the association phase.

According to the Bayesian causal inference model (Körding et al., 2007), changes in either the unimodal reliabilities or the crossmodal causal prior could alter the degree of multisensory integration. Unimodal localization performance did not differ significantly between the Pre- and Post-tests for A1, A2, V1, or V2. Specifically, for each unimodal stimulus, neither the Variable Error, which is inversely proportional to reliability, nor the Constant Error, which is a measure of systematic spatial bias or localization accuracy, differed before and after the association phase. This supports the notion that the observed VE differences were unlikely to be driven by changes in the unimodal reliabilities or localization accuracy. Taken together with the AV test results, this suggests that association pairing was effective in setting up causal priors: crossmodal binding tendencies were higher following experience with co-occurring A and V stimuli relative to experience with discrepant A and V stimuli. Moreover, this effect should be more apparent at large spatial disparities, where the absolute shift in auditory localization is maximal at approximately 20° (Bertelson & Radeau, 1981; Hairston et al., 2003; Wallace et al., 2004).

Interestingly, the observed VE did not differ significantly between the stimulus pairs involving A1 (i.e., A1V1 and A1V2) or those involving A2 (i.e., A2V1 and A2V2), but it differed significantly between the recombined pairs A1V2 and A2V1. In other words, the size of the VE, and by extension the AV binding, seemed to be determined by the auditory stimulus that signified congruent or incongruent AV relations and generalized over the visual components. Importantly, the counterbalanced design of the tones used for congruent (A1) versus incongruent (A2) stimuli ruled out the possibility that this effect was simply due to the salience of particular tones. Indeed, unimodal reliabilities did not differ between A1 and A2 in the Pre- and Post-tests. Taken together, our results suggest that association pairing produced different levels of AV binding for stimulus pairs that included A1 versus A2. A couple of reasons might explain why participants relied on the auditory stimulus to determine when it was appropriate to integrate it with a visual stimulus in our experimental setup. First, participants were explicitly instructed to focus on localizing the tone and to ignore the flash on AV trials. Thus, participants might have been motivated to pay closer attention to the auditory feature (frequency) over the visual feature (color); this might have contributed to the generalization of the auditory-driven effects over visual stimuli. Second, it was likely more difficult to discriminate the colors (red and blue) than the tones (750 and 3,000 Hz). Color discrimination varies with retinal location and diminishes towards the periphery, a finding well supported by both behavioral and physiological studies (Mullen & Kingdom, 1996; Newton & Eskew, 2003), whereas sound frequency discrimination depends less on eccentricity.

A previous study (Odegaard et al., 2017) examined what types of spatiotemporal relations between AV stimuli were able to change subsequent AV binding tendencies. In a between-subjects design, different groups of participants were exposed to six different spatiotemporal congruency conditions of AV stimuli. After the association phase, the spatial VE was measured as an index for the degree of AV integration. Surprisingly, the authors reported that the most effective method to increase subsequent AV binding was an exposure to temporally congruent but spatially incongruent AV pairs. These results were surprising because the literature generally demonstrated the crucial role of spatiotemporal coincidence for associating stimuli with the same cause (e.g., Chen & Spence, 2017; Rohe & Noppeney, 2015b; Wallace et al., 2004). Unlike our findings, Odegaard et al. did not observe an increase in subsequent multisensory binding for AV pairs that were both spatially and temporally congruent, or a decrease in subsequent multisensory binding for AV pairs that were both spatially and temporally incongruent.

In contrast to Odegaard et al., a key design feature in our experiment was that participants were exposed to spatiotemporally congruent *and* incongruent pairs in parallel during the association phase, and the pairs were later tested in mixed-trials blocks. We reasoned that if parallel exposure to two distinct pairs of AV stimuli with obviously distinct spatiotemporal relations would result in significantly different degrees of subsequent AV integration, such a finding would suggest that the pairing manipulations had differential impacts on priors. Moreover, by attempting to increase the prior for one stimulus pair while in parallel decreasing the prior for the other pair, we minimized potential ceiling or floor effects on the causal priors. In addition, our mixed presentation of congruent and incongruent stimuli reflects common characteristics of natural stimulus statistics, where subsets of experienced crossmodal inputs are congruent while others are incongruent. Furthermore, we presented our stimulus pairs across a much larger range of locations than those used in Odegaard et al., which might help generalize the learned associative or dissociative relations of auditory and visual stimuli across space, rendering the learning more effective.

Another difference between our study and that of Odegaard et al. is that they presented their temporally incongruent stimuli with SOAs randomly sampled in the range of −250–250 ms. The temporal binding window for AV stimuli can be as high as 200–300 ms (Wallace & Stevenson, 2014), that is, AV pairs with short SOAs within this temporal window may be perceptually combined similarly to simultaneous AV pairs. Thus, it is possible that many of Odegaard et al.’s temporally incongruent pairs appeared synchronous to the participants. By contrast, we chose longer SOAs (randomly sampled in the range of 750–1,500 ms) that were much greater than the AV temporal binding windows and thus would presumably give rise to the perception of distinctly asynchronous stimuli. We speculate that the perception of distinct events in time is crucial for the formation of priors that such events belong to different causes. Alternatively, it might be suggested that participants had learned A2 and V2 not as a dissociated pair but rather as separate unisensory events; therefore, the learned priors could be that A2 and V2 were in general not linked to any of the remaining stimuli. A similar argument could be made about A1 and V1: participants might have updated their priors regarding A1 and V1 not as an associated pair but rather as distinct unimodal stimuli that were associated with a presentation in the other modality. To investigate the nature of the pairing effects, Experiment 2 was conducted.

## Experiment 2

Experiment 1 demonstrated that the VE changed as a consequence of manipulating the belief about whether an A and a V belong to the same event or different events. In Experiment 1, we observed a generalization of the auditory stimulus history (association or dissociation with a visual stimulus) across visual stimuli, that is, the VE did not differ for A1V1 and A1V2, nor for A2V2 and A2V1. Experiment 2 explored the specificity and flexibility of association learning in modifying multisensory binding. In Experiment 2, participants were exposed to the same association procedure as in Experiment 1; however, the crucial new manipulation here was that during the audiovisual Test blocks, the auditory stimulus was presented simultaneously with two competing visual stimuli flanking its sides, instead of just one visual stimulus. If specific AV spatiotemporal histories were learned, then presenting the A1 between the V1 and V2 should result in a VE towards the V1; if no specific AV spatiotemporal histories were learned, then no VE should be observed because the V1 and V2 would equally attract the perceived A location. Furthermore, a new auditory stimulus (A3), never before presented in the Association blocks, was additionally tested to explore whether the history of the visual stimuli would generalize to a novel auditory stimulus.

### Methods

#### Participants

We recruited a new set of 24 participants from the University of Hamburg community. One participant was later disqualified due to poor performance on the Association blocks (see Association blocks section); data reported were based on the remaining 23 participants (ages: 18–54 years, median age = 24 years, interquartile range = 21–25.25 years), only one individual was over 45 years old; 22 were right-handed, 14 female). The ethics approval, informed consent, pre-screening, handedness test, and reimbursement procedures were the same as for Experiment 1.

#### Apparatus and stimuli

The testing apparatus and stimuli were identical to those used in Experiment 1 (see Experiment 1, Methods – Apparatus and stimuli) except for the addition of a novel auditory stimulus A3 (1,500 Hz sine tone) to the Pre-test, Post-test, and AV Test blocks. A3 was not presented during the Association blocks. As in Experiment 1, the auditory (A1, A2) and visual (V1, V2) stimuli used in the congruent versus incongruent stimulus pair were counterbalanced across participants and consistent for each individual participant.

#### Testing procedure

Each participant completed one experimental session (Fig. 1, Experiment 2). Participants first completed a Pre-test measuring their unisensory auditory and visual localization performance. This was followed by three alternating Association blocks and AV Test blocks. The experiment then ended with a unimodal Post-test, which was identical to the Pre-test. The session lasted 2.5–3 h, including short (2–3 min) breaks roughly every 30 min.

##### Unimodal Pre- and Post-tests

These were identical to those in Experiment 1 except for the addition of A3 (Fig. 2a).

##### Association blocks

These were also identical to those in Experiment 1 (Fig. 2b).

##### Audiovisual Test blocks (Fig. 2d)

As in Experiment 1, the task was auditory localization: Participants were explicitly asked to pay attention to the auditory (A) stimulus while maintaining fixation and to respond by localizing only the A stimulus. Following the offset of a 1,000-ms fixation-point and a delay randomly varying between 825 and 1,025 ms, one of the three possible A stimuli was presented simultaneously with two visual (V) stimuli flanking the A at equal distances, yielding three test stimulus triplets: V1A1V2, V1A2V2, and V1A3V2. For example, if V1 was presented 4.5° to the left of A, then V2 would be presented 4.5° to the right of A. The positions of V1 and V2 relative to A (i.e., the visual configurations) were balanced: the number of trials in which V1 was to the left of A was equal to the number of trials in which V1 was to the right of A. This was done for each of the A stimuli (A1, A2, and A3) at each of four speaker positions (± 4.5° and ± 13.5°) with AV disparities of ± 4.5°, ± 9°, and ± 18°. Therefore, there were a total of 72 unique stimulus conditions (three auditory stimuli × four speaker locations × three disparities × two visual configurations); each of these stimulus combinations was tested 12 times for a total of 864 test trials. In addition to these test trials, 20 deviant trials were added to each block to ensure participants kept their eyes open. The deviant trials were identical to those used in Experiment 1: participants pressed either of two buttons when they saw the fixation-point blinking. The deviant trials were later excluded from data analysis.

#### Data analysis

Statistical analyses for the unimodal Pre- and Post-tests and the association blocks were identical to those in Experiment 1 (see Experiment 1, Data analysis for detail). Additionally, for the post hoc pairwise comparisons, paired t-tests were conducted using the R-package “emmeans” and the *p*-values were corrected by the Bonferroni-Holm method.

##### Audiovisual Test blocks

To measure the extent of the overall VE, a VE index was derived for each absolute value of disparity. Unlike in Experiment 1, the audiovisual Test blocks in Experiment 2 presented two visual stimuli with positive and negative disparities on every trial; therefore, we redefined “disparity” as the spatial position of the congruent visual stimulus (V1) relative to the auditory stimulus; that is, disparity = V1 position – A position. For example, a disparity value of +4.5° means that V1 was to the right of A by 4.5°, while a disparity value of −4.5° means that V1 was to the left of A by 4.5°. With this definition of disparity, the VE index values were then calculated in a similar manner as in Experiment 1: the mean localization value for the negative-disparity trials (i.e., V1 on the left and V2 on the right of A) was subtracted from that of the positive-disparity trials (i.e., V2 on the left and V1 on the right of A) of the same absolute disparity value. Thus, a positive VE index value indicates a net shift in perceived auditory location towards V1, whereas a negative VE index value indicates a net shift towards V2 (i.e., away from V1). These VE index values were then averaged over speaker positions to provide a summary of the strength of the VE for each combination of absolute disparity and stimulus triplet (V1A1V2, V1A2V2, V1A3V2) for each participant. The VE index values were analyzed with a 3 × 3 ANOVA, with absolute Disparity (4.5°, 9°, and 18°) and Auditory stimulus (A1, A2, and A3) as factors.

Given that there was a significant effect of Auditory stimulus on the level of VE measured, we carried out pairwise t-tests between the different auditory stimuli (A1, A2, and A3) to identify the exact pairwise differences in sound localization linked to association pairing.

### Results

#### Unimodal Pre- and Post-tests

The mean Variable Errors and Constant Errors of unimodal localization performance in the Pre- and Post-tests are plotted in Fig. S3, Supplemental Online Material. On Variable Errors, in the auditory modality, there was a significant main effect of Measuring Time (Pre-test, Post-test): the mean auditory Variable Errors over all auditory stimuli (A1, A2, and A3) were higher in the Post-test than in the Pre-test (F(1,22) = 6.280, p = .020, η_G_^2^ = .036). However, no significant main effects of Stimulus Type (A1, A2, and A3 for auditory, V1 and V2 for visual) or interactions between Stimulus Type and Measuring Time were observed in either modality; moreover, there was no significant main effect of Measuring Time for the visual Pre- versus Post-tests. On Constant Errors, in either modality, no significant main effects of Measuring Time, Stimulus Type, or interactions between these factors were observed (all ps ≥ .066; see Table S1 for details).

Although the overall auditory Variable Errors were higher (indicating lower auditory reliabilities) in the Post-test compared to the Pre-test, there was no significant main effect of Stimulus Type and, more importantly, no significant interaction between Stimulus Type and Measurement Time. Therefore, we conclude that the association pairing manipulations did not differentially change the reliabilities of the unimodal component stimuli.

#### Association blocks

The average percent correct on a total of 144 one-back trials across all three Association blocks was calculated. All but one participant was able to perform the task above chance level (>50%; median performance 86%), suggesting that most participants could reliably discriminate the color of the lasers. This disqualified participant performed below our criterion of 60%, with an accuracy level of 44%; all data of this participant were excluded from analyses.

#### Audiovisual Test blocks

**Fig. 4:**
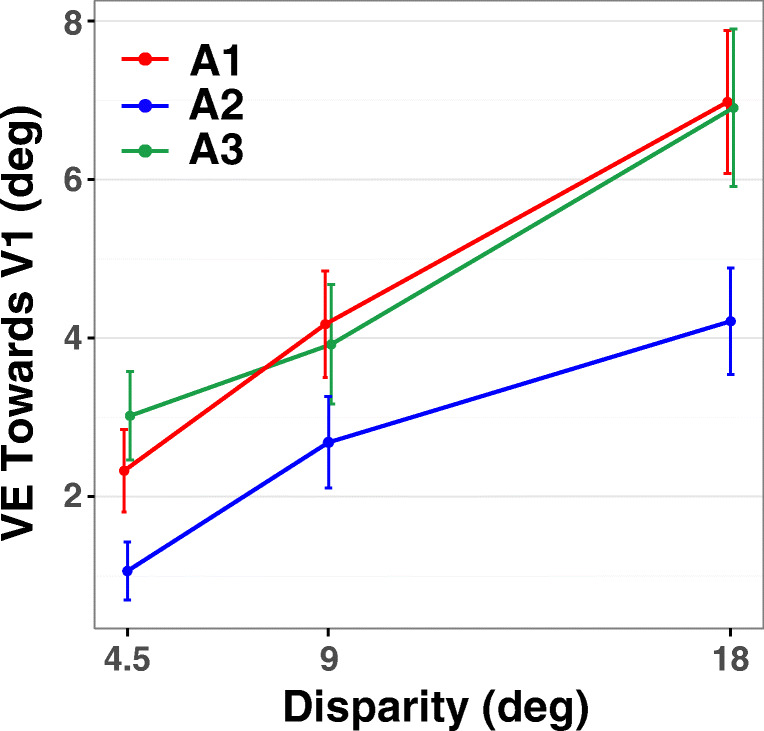
Ventriloquism effect (VE) towards V1 (Experiment 2). VE index towards V1 (previously presented in the congruent audiovisual pair during the association phase), plotted as a function of audiovisual disparity. This index represents a net shift of auditory localization towards V1 when a pair of competing visual stimuli (V1 and V2) symmetrically and simultaneously flanked the auditory stimulus during test trials. Different colors represent different auditory stimuli tested: red = A1 (previously congruent), blue = A2 (previously incongruent), green = A3 (not presented during association phase). Lines are slightly dodged horizontally to reduce overlap for illustration purpose. Error bars: ± 1 SEM

If the competing visual stimuli equally affected the localization of the flanked auditory stimulus in the middle, these effects would cancel out and we would observe zero net VE. We observed, however, a positive VE index value for each auditory stimulus. The VE index values averaged across disparities were (mean ± 1 standard deviation): 4.49 ± 2.77° for A1, 2.65 ± 2.03° for A2, and 4.61 ± 3.03° for A3. One-tailed t-tests confirmed that the VE index was indeed significantly greater than zero for all auditory stimuli (A1: t(22) = 7.775, p < .001, Cohen’s d = 1.621; A2: t(22) = 6.259, p < .001, d = 1.305; A3: t(22) = 7.285, p < .001, d = 1.519). These positive VE index values indicate that for all three AV stimulus triplets, on average, auditory localization shifted towards the congruent visual stimulus, V1 (Fig. 4). Repeated-measures ANOVA on the VE index values showed significant main effects of Auditory stimulus (F(2,44) = 10.624; p < .001, η_G_^2^ = .071) and Disparity (F(2,44) = 38.083; p < .001, ε = .736, η_G_^2^ = .197), but no significant interaction between Auditory stimulus and Disparity (F(4,88) = 1.001; p = .412, η_G_^2^ = .010). Post hoc pairwise comparisons (paired t-tests) indicated that the VE index values significantly differed between A1 and A2 (t(44) = 3.858, p < .001, d = .804), and between A3 and A2 (t(44) = 4.114, p < .001, d = .863), but not between A1 and A3 (t(44) = -.256, p = .799, d = .053).

### Discussion

Experiment 2 further investigated the effects of association learning, that is, the acquisition of priors for AV pairs that were spatiotemporally either congruent or incongruent. If an auditory stimulus was spatiotemporally either congruent or incongruent with a visual stimulus during an association phase, then in subsequent test trials where different visual stimuli were simultaneously presented with the auditory stimulus, the visual stimulus towards which the auditory stimulus was mislocalized can reveal the special causal priors formed for different AV pairs. Results of Experiment 2 indicated that auditory localization indeed shifted towards V1. This effect was observed for all three auditory stimuli, including the new auditory stimulus (A3) that had never been presented during the association phase. Interestingly, the amount of VE observed for A1V1 was generalized to A3 (Fig. 4).

These results support and extend the findings of Experiment 1. First, both Experiments 1 and 2 showed that congruent pairing led to greater subsequent AV binding than incongruent pairing did. Second, this differential impact depended on the task and the stimulus context; the changes in AV binding were stimulus-specific but accompanied with some degree of generalization as well. In Experiment 1 the generalization occurred across visual stimuli, whereas in Experiment 2 the generalization occurred across auditory stimuli when a new sound was presented. These different patterns of generalization might point to the factors which guide multisensory integration in different contexts: In Experiment 1, there was at most one single flash presented with the sound, whereas in Experiment 2, two equally salient flashes competed for AV integration. The identity of the sound alone was informative for determining whether it was appropriate to integrate the sound with a visual stimulus, but not sufficient for determining *which* visual stimulus to integrate if there were competing visual stimuli. In the latter case, the colors of the visual stimuli that signified the congruent or incongruent status might become the primary determinant of the winning visual attractor, and this effect may generalize to a new sound. These findings are consistent with previous research showing that adults’ ability in differentiating congruent versus recombined AV pairs depend largely on stimulus context and task relevance (Rohlf, Habets, von Frieling, & Röder, 2017).

Importantly, we found that the shift in the perceived location towards V1 was smaller for A2 (incongruent) than for A1 (congruent) and for A3 (new). A2 was frequently presented during the Association blocks but never occurred together with V1; we speculate that this lack of co-occurrence might be taken as evidence for an unrelatedness between A2 and V1. Consequently, A2 might become partially decorrelated from V1. This decorrelation was likely less prominent than a decorrelation from the incongruent V2, since A2 was not presented in spatiotemporal asynchrony with V1 as it was with V2. As a result, A2 shifted away from V2 more than from V1. Regardless of whether the observed shifts were driven by learned association or dissociation, it is worth noting that they both must reflect a modulation of causal priors. Furthermore, the observed VE patterns suggest that this modulation of the causal prior at least partially took into account the specific association or dissociation between two stimuli, instead of only whether a unimodal cue was paired with any stimulus in the other modality (see the last paragraph of Experiment 1, Discussion). If the latter was the case, then A1 would have been equally linked to V1 and V2, and A2 would have been equally decorrelated from V1 and V2, both of which would have resulted in no shift in auditory localization. Similarly, if the effects were driven by modulated priors about the unimodal visual instead of auditory cues, then V1 would have been equally linked to A1 and A2, and V2 would have been equally decorrelated from A1 and A2, and these would have resulted in similar shifts towards V1 for A1 and A2. We did not observe either of these patterns.

Bertelson, Vroomen, De Gelder, and Driver (2000) employed a similar stimulus presentation to ours in Experiment 2: simple sound stimuli (tone bursts) were presented synchronously with two visual stimuli (squares) bilaterally flanking the sound with a constant disparity. However, unlike the present study, their goal was to determine whether spatial attention could bias the VE toward one visual stimulus or another. The authors showed that having identical flanking visual stimuli eliminated the VE and that thus the VE was independent of spatial attention. By contrast, manipulating the relative physical saliency of the visual stimuli elicited a VE toward the more salient visual stimulus. However, our experimental design ruled out differences in visual stimulus saliency as the explanation for the observed pattern of VE: the specific stimuli used as the congruent and incongruent stimulus components (V1 vs. V2, A1 vs. A2) were counterbalanced across participants. Moreover, the unimodal measurements of reliability (Variable Error) or accuracy (Constant Error) did not differ between the types of visual (V1, V2) or auditory (A1, A2) stimuli in both the Pre- and the Post-tests, suggesting that the changes in the VE could not be due to changes in unisensory processing. Therefore, the present associative pairing altered the subsequent AV integration via stimulus-specific modification of multisensory binding.

## General discussion

Here, we carried out two experiments to investigate whether it was possible to flexibly update the prior for a common cause (i.e., the causal prior) in opposite directions simultaneously for different pairs of AV stimuli. During Association blocks, participants were repeatedly exposed to spatiotemporally congruent and incongruent AV stimulus pairs. We predicted that following the Association blocks the prior would be greater for the congruent AV stimuli than for the incongruent ones, thereby driving the extent of subsequent AV integration and thus the size of VE to diverge. Indeed, the present experiments support these predictions: Following the association phase, greater VE was observed for spatiotemporally congruent stimuli (A1V1) than for incongruent stimuli (A2V2), even though the unimodal localization reliabilities of the individual auditory or visual components did not change. Moreover, we found that in a classic VE paradigm where AV stimuli were tested pairwise, the effects of updated causal priors on integration were largely determined by the auditory stimulus and generalized over visual stimuli: stimulus pairs including A1 (A1V1 and A1V2) consistently showed greater VE than those including A2 (A2V2 and A2V1) (Experiment 1). However, when two visual attractors competed for the perceived sound location, visual stimulus features influenced the degree of integration (Experiment 2). The findings agree with the hypotheses and predictions of the Bayesian inference framework: crossmodal causal priors, which determine multisensory binding tendencies, adapt to the statistics in the environment with a great amount of flexibility.

A central focus of multisensory research is the influence of top-down factors on the binding or integration of multiple sensory cues. In contrast to bottom-up factors of multisensory processing, such as perceptual salience or present spatiotemporal coincidence of the stimuli, top-down factors consider higher-order abstractions and reflect the internal states of the brain, such as attention, semantic congruency, and crossmodal correspondence (for reviews, see Chen & Vroomen 2013; Chen & Spence, 2017). In the Bayesian perceptual framework, bottom-up factors can be quantified by likelihood distributions, while top-down factors can be summarized as prior probabilities. Here, we have provided evidence that priors can be updated by using simple stimuli in an association-pairing procedure, where adaptation to intermixed spatiotemporal congruence and incongruence is capable of producing different levels of subsequent multisensory integration. We chose to use simple stimuli – sine tones of different frequencies and flashes of different colors – in order to minimize any semantic associations. Our intention was to have participants rely on simple bottom-up factors of spatiotemporal alignment to form associations between particular pairs of stimuli, which in turn would be subsequently used as top-down factors for AV integration during a VE paradigm.

Our study contributes to the growing literature investigating the adaptability of crossmodal correspondence and its impacts on multisensory perception (for a review, see Spence, 2011). For example, Ernst (2007) demonstrated that exposure to arbitrary correspondence between the luminance of a visual object and its haptic stiffness – stimulus dimensions that are uncorrelated in the natural environment – can artificially induce correlations between these stimulus dimensions. Moreover, observers use cue correlations to infer causation. For example, temporally correlated auditory and visual signals are inferred to originate from the same event and hence integrated optimally (Parise et al., 2012). The prior of a common cause has been shown to be positively correlated with the degree of multisensory integration, whether such prior is experimentally induced (Odegaard & Shams, 2016) or mirrors natural correlations among stimulus features (Ernst, 2006).

Although we have interpreted our findings thus far with the notion that both association and dissociation during the training phase caused the observed differences in VE, and that any lack of differences may be due to generalizations, we should consider the alternative possibility that dissociation was the primary driving factor. With this interpretation there exists a high baseline for integration of synchronous AV stimuli, which may explain the robustness of VE with arbitrary AV pairs. However, when presented with strong evidence for the spatiotemporal unrelatedness of certain AV stimuli (e.g., A2V2 and A2V1), observers lower the binding tendencies for such AV stimuli. In Experiment 1, the comparable sizes of VE for A1V1 and A1V2 might both reflect a near baseline level of prior and AV integration, whereas the smaller VE for both A2V2 and A2V1 might reflect a reduced prior and degree of integration. Although A1V2 and A2V1 were both unpaired during the association phase and thus should have been decorrelated, the pattern of effects might be primarily dependent on the auditory stimulus and generalized across visual stimuli, for reasons discussed above in Experiment 1, Discussion. In Experiment 2, neither A1 nor A3 had been previously paired with V2, and therefore they were decorrelated from and hence less integrated with V2. Consequently, they were pulled towards V1 because of the baseline level of integration between them and V1. It is plausible that there exists a moderately strong baseline tendency for AV integration in general, which would explain why the VE is ubiquitous in daily life even for arbitrary stimuli. We would generalize across stimuli with this baseline tendency for integration, unless when the environment or task requires more specificity. The presentation of AV stimuli being misaligned or uncorrelated in both space and time might provide a strong cue for such a need, thus causing a decrease in integration.

Our results partially support the Bayesian causal inference model of multisensory integration, but an all-encompassing causal prior cannot fully explain either the generalization of effects to other auditory or visual stimuli, or why this generalization depends on the specific task context. It is possible that observers learn priors about associations between stimulus pairs as well as about unimodal cues. Experiment 2 partially addressed this issue, but it remains unclear how the priors were updated with respect to cue associations versus individual cues, and whether multiple causal priors contribute to crossmodal binding. Future studies should include an AV pre-association test to measure the baseline level of integration in order to draw definite conclusions about whether the integration in fact increased or decreased through exposure to association/dissociation trials. Future studies could also directly probe the tendency to judge crossmodal stimuli as sharing a common cause by directly asking participants on each trial whether they perceived the stimuli as being unified. In addition, the Bayesian causal inference model could be extended to account for the generalization patterns that we observed.

Another future direction is to unravel the neural correlates of association learning and causal inference. Several studies have documented neural activity changes accompanying the acquisition of AV associations or dissociations through exposure to repeated pairing for just a few tens of minutes (e.g., Baier, Kleinschmidt, & Muller, 2006; Fiebelkorn, Foxe, & Molholm, 2010; Zangenehpour & Zatorre, 2010); this association learning seems to optimize functional connectivity between specialized cortical sensory regions and thus might facilitate object recognition (von Kriegstein & Giraud, 2006). A recent fMRI study found evidence for the causal inference model (Körding et al., 2007) by identifying brain regions with activation patterns that correlated with estimates produced by the model’s computational components (Rohe & Noppeney, 2015a). However, how neural circuits implement priors for multisensory binding is not yet known. It is plausible that the representation of priors is distributed throughout association cortices and not necessarily localized to a specific brain region. One proposal is that priors may be encoded in the spontaneous activity of neurons (Berkes, Orbán, Lengyel, & Fiser, 2011). Moreover, top-down feedback projections to early sensory cortices seem to be involved in the learning of priors, and this has been demonstrated in infants as young as 6 months of age (Emberson, Richards, & Aslin, 2015). Future research could use a combination of behavioral studies, brain-imaging techniques, and neural network models (e.g., Ursino, Crisafulli, di Pellegrino, Magosso, & Cuppini, 2017) to help shed light on the neural correlates and computational principles that underlie the learning of new causal priors and its effects on multisensory binding.

## Conclusion

We demonstrated that audiovisual (AV) stimuli paired to be spatiotemporally congruent showed higher subsequent spatial integration than AV stimuli paired to be spatiotemporally incongruent, and that the generalizability of this effect depended on the stimulus context. Since multisensory binding tendencies can be characterized by causal priors linking crossmodal stimuli, our main results support the Bayesian causal inference framework of multisensory integration. However, how Bayesian causal inference models handle generalization effects as observed in the present study needs to be investigated. The present results suggest that multisensory binding is flexible and dynamically adaptive to the changing crossmodal statistics in the environment.

## Electronic supplementary material

ESM 1 (DOCX 1261 kb)
