# Supplementary material for: Crossmodal associations modulate multisensory spatial integration
Source: Atten Percept Psychophys. 2020 Jul 5;82(7):3490–506. doi: 10.3758/s13414-020-02083-2 (PMC7536156; doi:10.3758/s13414-020-02083-2)
Supplement: Supplementary file 1 — (DOCX 1261 kb) [file 13414_2020_2083_MOESM1_ESM.docx]

**Supplementary Material**


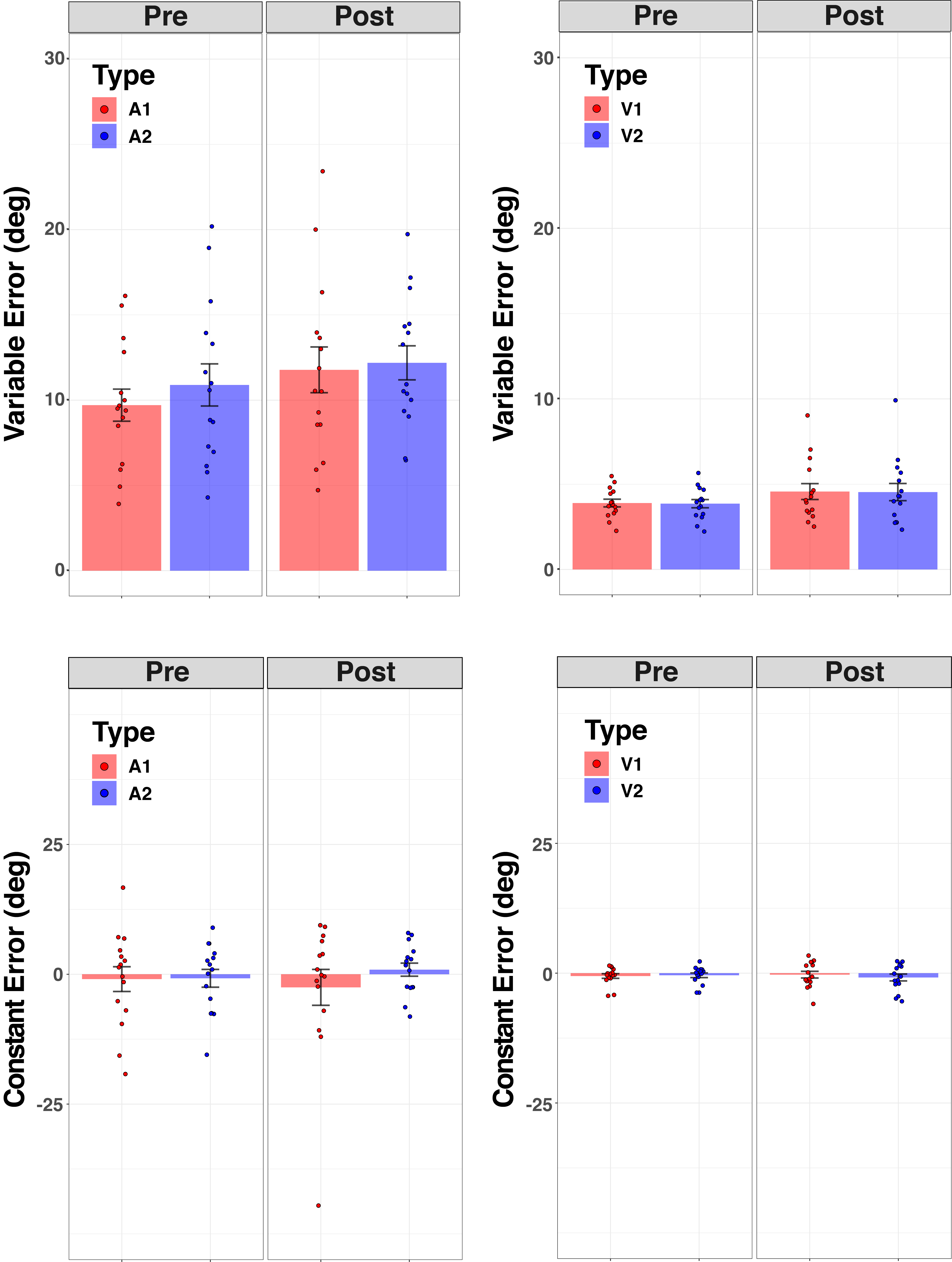


**Figure S1.** **Pre- and Post-test measurements of Variable Error and Constant Error (Experiment 1)**. **Top row:** Variable Error. **Left**: Pre- and Post-test mean Variable Errors for the auditory stimuli used in congruent (red, A1) and incongruent (blue, A2) audiovisual pairing. **Right**: Pre- and Post-test mean Variable Error for the visual stimuli used in congruent (red, V1) and incongruent (blue, V2) audiovisual pairing.

**Bottom row:** Constant Error. **Left**: Pre- and Post-test mean Constant Errors for the auditory stimuli used in congruent (red, A1) and incongruent (blue, A2) audiovisual pairing. **Right**: Pre- and Post-test mean Constant Error for the visual stimuli used in congruent (red, V1) and incongruent (blue, V2) audiovisual pairing.

Dots: individual participants; for illustration, data points are slightly dodged horizontally to reduce overlap. Bars: condition means. Error bars: ±1 SEM.

**Table S1. Pre- and Post-tests ANOVA results for unimodal Variable Error and Constant Error (Experiments 1^†^ and 2^††^).**

| **Experiment 1** | | | | | | |
| --- | --- | --- | --- | --- | --- | --- |
| **Variable Error** | **Auditory** | | | **Visual** | | |
|  | F(1,14) | p | η_G_^2^ | F(1,14) | p | η_G_^2^ |
| Stimulus Type | .666 | .428 | .009 | .0482 | .829 | < .001 |
| Measurement Time | 2.417 | .142 | .037 | 2.749 | .119 | .053 |
| Stimulus Type:Measurement Time | .340 | .569 | .002 | .0002 | .988 | < .001 |
| **Constant Error** | **Auditory** | | | **Visual** | | |
|  | F(1,14) | p | η_G_^2^ | F(1,14) | p | η_G_^2^ |
| Stimulus Type | .459 | .509 | .010 | .818 | .381 | .002 |
| Measurement Time | .0004 | .983 | < .001 | .034 | .856 | < .001 |
| Stimulus Type:Measurement Time | 1.069 | .319 | .008 | 3.655 | .077 | .007 |

| **Experiment 2** | | | | | | |
| --- | --- | --- | --- | --- | --- | --- |
| **Variable Error** | **Auditory** | | | **Visual** | | |
|  | F | p | η_G_^2^ | F | p | η_G_^2^ |
| Stimulus Type | F(2,44)= 2.045 | .142 | .020 | F(2,44)=  .044 | .836 | < .001 |
| Measurement Time | F(1,22)= 6.280 | .020* | .036 | F(1,22)= .372 | .548 | .004 |
| Stimulus Type:Measurement Time | F(2,44)=  .282 | .755 | < .001 | F(2,44)= .106 | .748 | < .001 |
| **Constant Error** | **Auditory** | | | **Visual** | | |
|  | F | p | η_G_^2^ | F | p | η_G_^2^ |
| Stimulus Type | F(2,44)= 1.021 | .368 | .010 | F(2,44)= 1.438 | .243 | .002 |
| Measurement Time | F(1,22)= 1.084 | .309 | .007 | F(1,22)= 1.096 | .306 | .018 |
| Stimulus Type:Measurement Time | F(2,44)=  .397 | .675 | .002 | F(2,44)= 3.753 | .066 | .007 |

**^†^** Experiment 1. Stimulus type: A1, A2 for the auditory stimulus, and V1, V2 for the visual stimulus. Measurement time: Pre-test, Post-test.

**^††^** Experiment 2. Stimulus type: A1, A2, A3 for the auditory stimulus, and V1, V2 for the visual stimulus. Measurement time: Pre-test, Post-test.

* Significance level: .05.

**Figure S2. Constant Error as a function of audiovisual (AV) disparity for the AV Test blocks of Experiment 1.** The mean Constant Error (i.e., the perceived auditory location minus the speaker location) as a function of AV disparity, plotted for every speaker location, stimulus pair, and SOA condition. Different colors represent different stimulus pairs: red = A1V1 (congruent), dark grey = A1V2 (recombined), light grey = A2V1 (recombined), blue = A2V2 (incongruent). Each row represents localization data for a given SOA condition (from top to bottom: -150, 0 and 150 ms; negative SOA indicates auditory first, positive SOA indicates visual first). Each column represents localization data at a given speaker location (from left to right: -13.5, -4.5, 4.5 and 13.5 deg). The slopes of the lines give a rough indication of the strength of the ventriloquism effect.


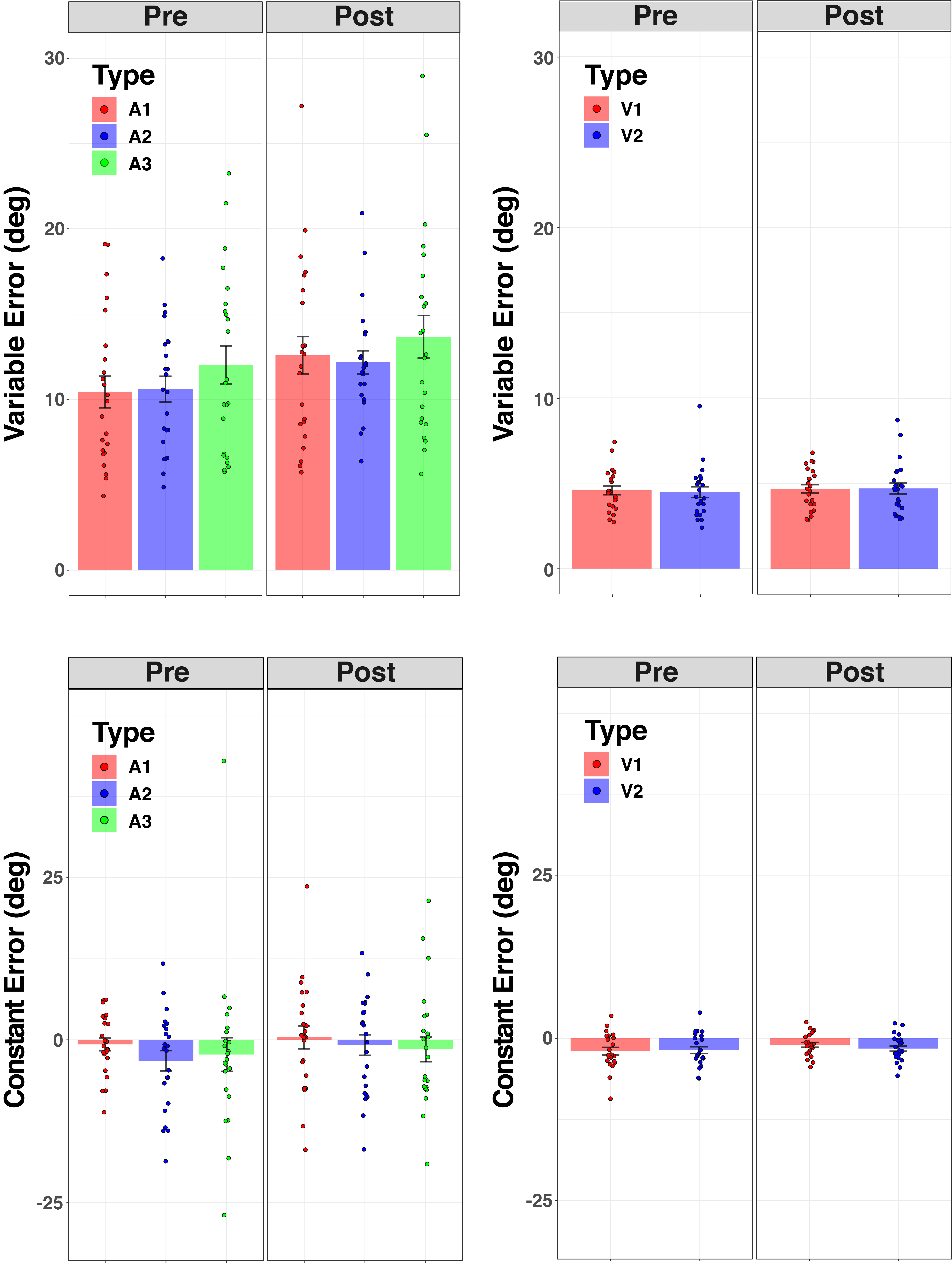


**Figure S3.** **Pre- and Post-test measurements of Variable Error and Constant Error (Experiment 2)**. **Top row:** Variable Error. **Left**: Pre- and Post-test mean Variable Errors for the auditory stimuli used in congruent (red, A1) and incongruent (blue, A2) audiovisual pairing, and for the new auditory stimulus (green, A3). **Right**: Pre- and Post-test mean Variable Error for the visual stimuli used in congruent (red, V1) and incongruent (blue, V2) audiovisual pairing.

**Bottom row:** Constant Error. **Left**: Pre- and Post-test mean Constant Errors for the auditory stimuli used in congruent (red, A1) and incongruent (blue, A2) audiovisual pairing, and for the new auditory stimulus (green, A3). **Right**: Pre- and Post-test mean Constant Error for the visual stimuli used in congruent (red, V1) and incongruent (blue, V2) audiovisual pairing.

Dots: individual participants; for illustration, data points are slightly dodged horizontally to reduce overlap. Bars: condition means. Error bars: ±1 SEM.
